# Influence of Laser Treatment on End Notched Flexure Bonded Joints in Carbon Fiber Reinforced Polymer: Experimental and Numerical Results

**DOI:** 10.3390/ma15030910

**Published:** 2022-01-25

**Authors:** Luca Sorrentino, Gianluca Parodo, Sandro Turchetta

**Affiliations:** Department of Civil and Mechanical Engineering, University of Cassino and Southern Lazio, 03043 Cassino, Italy; sorrentino@unicas.it (L.S.); turchetta@unicas.it (S.T.)

**Keywords:** polymer-matrix composites (PMCs), adhesion, surface treatments

## Abstract

Surface pretreatment for bonding is one of the most important steps for the manufacturing of a reliable bonded joint. In this paper, the effectiveness of an innovative pretreatment by CO_2_ pulsed laser for bonding Carbon Fiber Reinforced Polymer (CFRP) was investigated. End Notched Flexure (ENF) specimens were made with different densities of laser treatment, and the respective fracture toughness was obtained through the Compliance-Based Beam Method (CBBM). Furthermore, a cohesive model for simulating debonding processes was illustrated, and the cohesive parameters were obtained by an inverse method. The achieved results represent a fundamental step for the development of a numerical model useful for the determination of laser texturing as a function of the applied local stress into the bonded joint.

## 1. Introduction

The aerospace and military industries more and more often require the manufacturing of complex geometry lightweight components with high specific strength and stiffness. In this context, the use of fibro reinforced polymeric composite materials, thanks to their peculiarities, play a fundamental role in achieving such performance. However, at present, it is not always possible to produce complex geometries with this type of material in a single production step; therefore. the trend is to manufacture an assembly consisting of n-parts of simpler geometry. Nevertheless, the designers must take the presence of joints into account as they may have critical points. Traditional jointing techniques, such as riveting and bolting, require the drilling of the parts: it follows that the reinforcement must be cut, reducing their performance. In addition, the presence of holes can generate intensification of stresses that can cause structural failure during the use of the component. As an alternative to traditional fastening, such as riveting and bolting, the development of high-performance structural adhesives has grown in recent decades. Since it is not necessary to drill the parts, the use of structural adhesives is indicated in the case of assembly of components in polymeric composite materials, reducing weight, avoiding the presence of corrosive problems, allowing to dampen the vibrations, and, consequently, the reduction of vibroacoustic activity [1,2,3,4]. The reliability of a bonded joint is closely related to the chemical compatibility between the substrates, the nature of the adhesive and adherends, the geometric configuration of the joint, the working conditions, and, in particular, the pre-treatment of the surfaces to be bonded, as shown in many works in the literature [5,6,7,8,9,10].

Generally, in industrial application, peel ply represents a widely used solution for realizing a repeatable roughness surface. However, the use of a release agents may be necessary to facilitate the peeling of the peel ply from the surface of the laminate. As a result, the release agents can migrate from the peel ply to the surface of the laminate, lowering the quality of the adhesion between the substrates [11]. For this reason, additional cleaning with abrasive methods could be necessary. Manual sanding and grit blasting are the most common abrasive processes adopted in the industrial field. They are quite easy to be carried out, but at the same time can show problems of tool wearing, additional contamination, low reproducibility, and health problems for the operators due to the dust production in these types of processes [12].

As alternative to abrasive methods, excimer and CO_2_ lasers can ablate the epoxy resin of the matrix in a selective way without damaging the fiber reinforcement [13]. In particular, the wavelength of excimer lasers on UV avoids thermal degradation of the composite laminate [14]. However, CO_2_ lasers can be more appropriate in the industrial field because of their higher productivity, higher wall plug efficiency, and treatment speed [15].

The probability of burning the Carbon Fiber Reinforced Polymer (CFRP) parts, inducing delamination phenomena, is increased with the use of a CO_2_ lasers because of their wavelength on IR [16]. In fact, the high conductivity of the reinforcement transmits the heat generated by the process from the surface to the bulk of the part [17]. In this way, the interface between the matrix and reinforcement is weakened, leading to the formation of an extensive Heat Affected Zone (HAZ) [18]. Consequently, the reliability of the bonded joint is strongly linked to the parameter of the laser process chosen during the design phase [19]. 

The presence of singularities does not allow the designer to easily use criteria based on maximum stress, which are generally present at the edges of bonded joints, in particular in the case of brittle adhesives [20]. In fact, singularities are points, in linear elastic analysis, where the value of stress tends to infinity, introducing problems of mesh sensitivity for Finite Elements Method (FEM) solutions [21]. Otherwise, it is possible to avoid damage approach developed on the maximum stress using energetic criteria. In the fracture mechanics approach, energetic analyses are carried out for the forecast of the damage evolution, and the main parameter used for this scope is the critical energy release rate (GC) that is obtainable directly from mechanical tests [22]. Under mode I and mode II loading, the fracture energy can be, respectively, determined using Double Cantilever Beam (DCB) and End Notched Flexure (ENF) tests [23,24].

Because of its simplicity, the Cohesive Zone Model (CZM) is widely used to describe the debonding process along a predefined path [25,26]. Here, the Fracture Process Zone (FPZ) is represented through two superimposed surfaces, and a traction-separation law between these two surfaces is adopted for the characterization of the debonding.

The shape of the cohesive law can influence the results of the simulations. Zhang et al. [27] observed that the shape of the cohesive law depended on the geometry of the joint and the type of adhesive. In particular, they experimented with butt-joints and DCB joints made with brittle and ductile adhesive. The results showed that the bilinear law was more suitable in the case of butt-joints made with brittle adhesive, while the trapezoidal law showed a better fitting with the results obtained from DCB joints made with ductile adhesive. In fact, the type of adhesive had particular influence on the mechanical resistance of a bonded joint [28].

At present, the pre-treatment for bonding in the industrial field does not take into account the stress distribution into the bonded joint. Because of this, the entire surface to be bonded is usually subjected to a pre-treatment made with the same process parameters, without taking into account the possible presence of singularity in the stress flied during the working life of the bonded joint. The presented paper represents a fundamental step to optimize the laser texturing of the surface to be bonded, taking into account the presence of singularities of stress. Specifically, this first research consisted of investigating the effectiveness of a laser texturing on the mechanical resistance of CFRP bonded joints under mode II. In particular, a cohesive model was used to predict the behavior of the specimens and a comparison between three cohesive laws was carried out for evaluating the most suitable law for modelling the investigated phenomenon.

## 2. Materials and Methods

**Figure 1:**
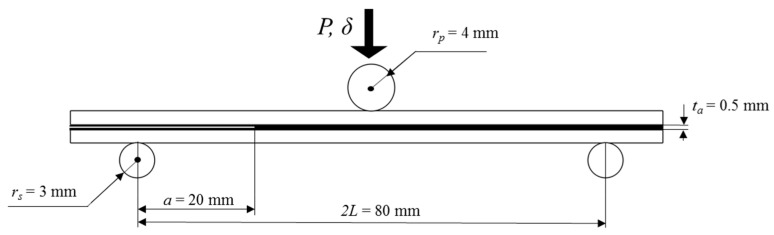
ENF specimen geometry and dimensions adopted in this work.

Figure 1 reports the ENF geometry and dimensions adopted for this work. The reference width was equal to 10 mm.

### 2.1. Experimental Activity

The laminates used for the production of ENF specimens were made by vacuum bagging molding using a unidirectional carbon fiber prepreg known as CYCOM T152/X751/135. The mechanical properties of the prepreg in the cured state are reported in Table 1. The layup chosen for the activity was a [0_2_/90/0_2_/90/$\bar{0}$]_s_. In this way, the manufactured adherends presented a thickness of 2.4 mm. After the polymerization and demolding of the laminates, they were polished using acetone to avoid the possible contamination of the surfaces with release agents. A CO_2_ Q-switched pulsed laser system with a peak power of 50 W was used for adherends pre-treating. The optical chain consisted of a laser source with a maximum average power of 25 W, a galvanometric mirrors system, a shutter, and a F-theta lens for focusing the laser beam. In particular, the process was made with a focal distance of 200 mm, while the spot size was 200 μm. The adopted texturing was based on dimples in a square grid, while the dimension of HAZ was limited minimizing the dimension of the dimples. The laser parameters for obtaining a repeatable dimple were achieved through a preliminary test and reported in Table 2.

**Figure 2:**
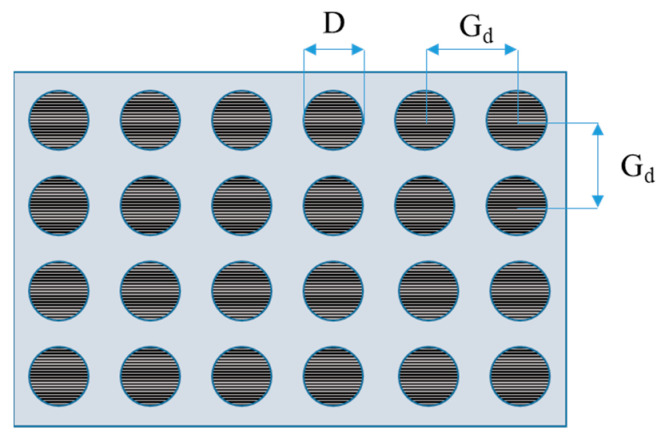
Texturing scheme adopted for the laser treatment: The texturing parameters were the dimples dimension (*D*) and the grid dimension (*G_d_*).

The adopted texturing allowed defining the density of treatment as a function of the only grid dimension. A sketch of texturing is reported in Figure 2.

In this analysis, the density of treatment is defined as:

(1) $$\rho =\frac{\pi D^{2}}{4G_{d}^{2}}\times 100$$

where *ρ* is the density of treatment, *G_d_* is the grid dimension of the texturing, and *D* is the dimension of the laser spot. Four levels of density were included in the experimental plan, as reported in Table 3. Preliminary analyses with optical microscope showed that no damage occurred on the CFRP laminate with the investigated values of densities of treatment.

In this work, the two-part epoxy paste adhesive EA 9309.3NA was used for manufacturing the ENF specimens. The bulk mechanical properties of the adhesive in the cured state are reported in Table 4. After 5 days from the application of the adhesive, the bonded laminates were demolded and subjected to cutting operation for obtaining the specimen with the nominal dimension (Figure 1).

Because the crack obtained from cutting was about 15 mm, a pre-cracking of 5 mm was made using a vise and a sharp blade for achieving the nominal value of 20 mm. Specifically, the specimens were gripped with the aid of rubber bands between the adherends and the vise: in this way, the damaging by compression of the CFRP was avoided. The position of the specimens in the vise was such that the crack tip before pre-cracking was 5 mm from the vise itself. A USB micro-camera was used to examine the crack propagation and stabilization at the target value, as shown in [30]. The specimens were tested in three-point bending tests using a universal machine with a crosshead speed of about 5.1 mm/min and a load cell of 10 kN. The compliance beam method (CBBM) was adopted for obtaining the fracture energy under mode II, presented in Equation (2), as shown in [28].

(2) $$G_{\mathrm{IIc}}=\frac{9P_{c}{}^{2}}{16b^{2}E_{f}h^{3}}{\left[\frac{C_{\mathrm{corr}}}{C_{0\mathrm{corr}}}a^{3}+\frac{2}{3}\left(\frac{C_{\mathrm{corr}}}{C_{0\mathrm{corr}}}-1\right)L^{3}\right]}^{2/3}$$

where *P_c_* is the failure load, a is the length of the crack before testing, *b* is the width, *E_f_* is the adherends flexural modulus, *h* is the adherends thickness, *L* is the span between punch and supports, and *C*_0corr_ and *C*_corr_ are:

(3) $$C_{0\mathrm{corr}}=C_{0}-\frac{3L}{10Gbh}$$

(4) $$C_{\mathrm{corr}}=C-\frac{3L}{10Gbh}$$

and represented, respectively, the initial specimen compliance and the corrected compliance, while *G* was the adherends shear modulus.

### 2.2. Numerical Model

**Figure 3:**
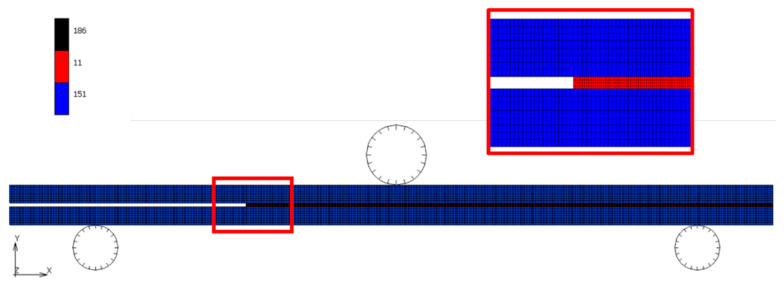
Geometry and mesh used for the simulations of ENF tests with related element types.

The commercial code MSC Marc/Mentat was used for modelling the increasing of ENF mechanical response due to the laser texturing. A linear elastic response of the CFRP and the epoxy adhesive in a 2D model was assumed. A mesh sensitivity analysis was carried out for defining the optimal number of the elements for the numerical model. From preliminary simulations, the size of the interface elements showed a low influence on the failure load. In fact, in cohesive models, the principal parameters used were the fracture energy G_I,c_ and G_II,c_, which were mesh-independent. However, the mesh size had to guarantee almost four elements into the fracture process zone, as stated in [31]. In total, 9600 four node isoparametric composite elements (type 151) and 3312 four node isoparametric isotropic elements (type 11) were used for modelling adherends and adhesive, respectively, as shown in Figure 3. In total, 414 interface elements (type 186) were realized with no thickness and located between adherend and adhesive elements. In this way, the model could simulate only adhesive failures. Indeed, the growth of the mechanical performance caused by laser texturing was quantifiable only if the flexural resistance of CFRP adherends was not exceeded, representing the limit condition.

**Figure 4:**
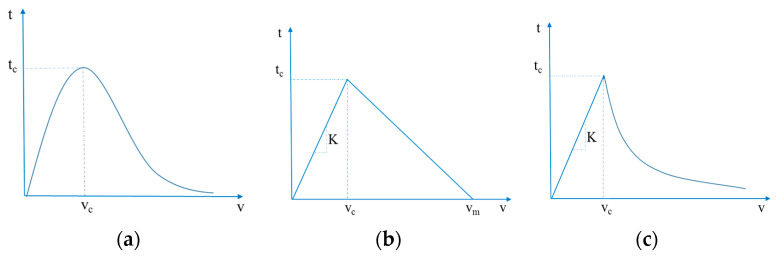
The shape of the cohesive laws used in this work: (**a**) exponential law; (**b**) bilinear law; (**c**) linear exponential law.

In this work, three types of cohesive traction-separation laws were investigated for modelling the ENF specimens: an exponential law, a bilinear law, and a linear-exponential law. In the exponential law, the traction can be defined as:

(5) $$t=\frac{G_{c}}{v_{c}^{2}}ve^{-\frac{v}{v_{c}}}$$

where *G_c_* is the critical energy release rate, *v_c_* is the critical displacement, and *t* and *v* are the stress and the displacement, respectively. In the bilinear law, the traction-separation law is equal to:

(6) $$t=\{\begin{array}{c} \frac{2G_{c}}{v_{m}v_{c}}v\;\;\;\;\;\;\;\;\;\;\;\;\;\;0\leq v\leq v_{c}\\ \frac{2G_{c}}{v_{m}}\left(\frac{v_{m}-v}{v_{m}-v_{c}}\right)\;\;\;\;\;\;v_{c}<v\leq v_{m}\\ 0\;\;\;\;\;\;\;\;\;\;\;\;\;\;\;\;\;\;v>v_{m} \end{array}$$

where *ν_m_* is the maximum displacement; and in the linear exponential law, the traction separation relation is defined as:

(7) $$t=\{\begin{array}{c} \frac{2qG_{c}}{v_{c}^{2}\left(2\;+\;q\right)}v\;\;\;\;\;\;\;\;\;\;\;\;\;\;\;0\leq v\leq v_{c}\\ \frac{2qG_{c}}{v_{c}^{2}\left(2\;+\;q\right)}e^{q\left(1-\frac{v}{v_{c}}\right)}\;\;\;\;\;\;v>v_{c} \end{array}$$

where *q* represents the exponential decay factor. A qualitative graphical representation of the cohesive laws adopted in this work is reported in Figure 4.

For the bilinear and the linear-exponential laws, it is possible to define the stiffness *K* as:

(8) $$K=\frac{t_{c}}{v_{c}}.$$

In this work, a quadratic stress criterion was used to predict the initiation of the damage:

(9) $${\left(\frac{t_{\mathrm{II}}}{t_{\mathrm{IIc}}}\right)}^{2}=1,$$

where *t*_II_ is the traction related to the pure mode II and *t*_IIc_ is the critical tractions under pure mode II. Instead, the crack growth was modelled with a linear energetic criterion:

(10) $$\frac{G_{\mathrm{II}}}{G_{\mathrm{II},c}}=1,$$

where *G*_II_ represents the area under the traction-separation law for pure mode II. The supports and the punch were modelled as rigid bodies through geometric entities, which were circles with the dimensions reported in Figure 1. The first boundary condition consisted of a fixed displacement along the x-axis of the nodes of the adherends under the center of the punch in a way to avoid rigid body moves. For the second boundary condition, the punch was subjected to a fixed velocity of −5.1 mm/min in the y direction.

From the experimental results, it was possible to obtain the fracture energy for the cohesive model, while the determination of the other cohesive parameters was achieved by an inverse method. Specifically, the evaluation of the other cohesive parameters for treated and untreated specimens was obtained by fitting the numerical load-displacement curves, as seen in [32].

## 3. Results and Discussions

### 3.1. Experimental Results

**Figure 5:**
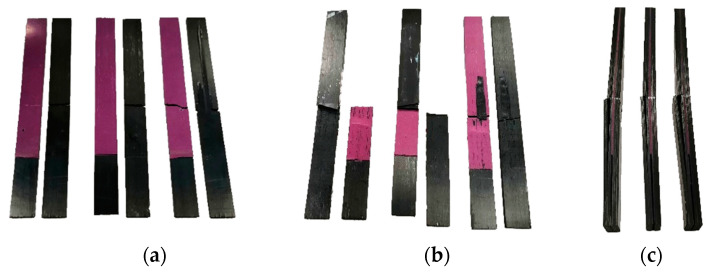
Inspection of ENF failure surfaces of: (**a**) untreated specimens; (**b**) 13% laser treated specimens; (**c**) 20% laser treated specimens.

To evaluate the contribution of the laser texturing to the mechanical resistance of the ENF specimens in a quantitative way, the failure had to happen at the adherend/adhesive interface. In fact, the increase of performance due to the laser treatment can be quantitatively evaluated only if the failure appears where the treated surface is located. For this purpose, the limit condition under which the failure in the specimens appeared at the interface was identified through experimental tests. The determination and classification of the failure modes was carried out according to ASTM D5573 [33]. Specifically, untreated specimens showed adhesive failures, while specimens with the density of treatment of 20% and 35% showed stock-break failures (Figure 5).

Specimens with the density of treatment of 13% showed more complex mixed failures, which were a combination between adhesive and stock-break failures. In particular, the failure appeared first as an adhesive type until the crack tip arrived in the middle of the specimens, subsequently as stock-break type with the bending failures of the adherends. For that reason, the density of treatment of 13% accounted for the limit condition, giving a mechanical resistance of the interface very close to the adherends delamination resistance. The maximum average load registered during testing showed the important effect of the laser treatment on the mechanical resistance of the ENF specimens. In particular, an average increase from 518.5 N to 1086.0 N was observed between untreated and 20% laser treated specimens (Table 5).

However, 20% laser treated specimens showed stock-break failures, so the obtained value did not represent the real increase of performance due to the laser treatment. Instead, an average increase from 518.5 N to 1040.4 N was observed between untreated and 13% laser treated specimens, more representative of the increase of performance due to the laser treatment. Specimens treated with a density of treatment of 35% showed a slight decrease of performance of about 3.6% respect specimens treated with a density of treatment of 20%. This could be due to a higher thermal degradation of the matrix of the first surface composite layer. However, observing the experimental range, it is possible to state that this variation is negligible. It is likely that densities of treatment higher than 35% could lead to thermal degradation of the matrix and, subsequently, a lower mechanical resistance of the adherends.

**Figure 6:**
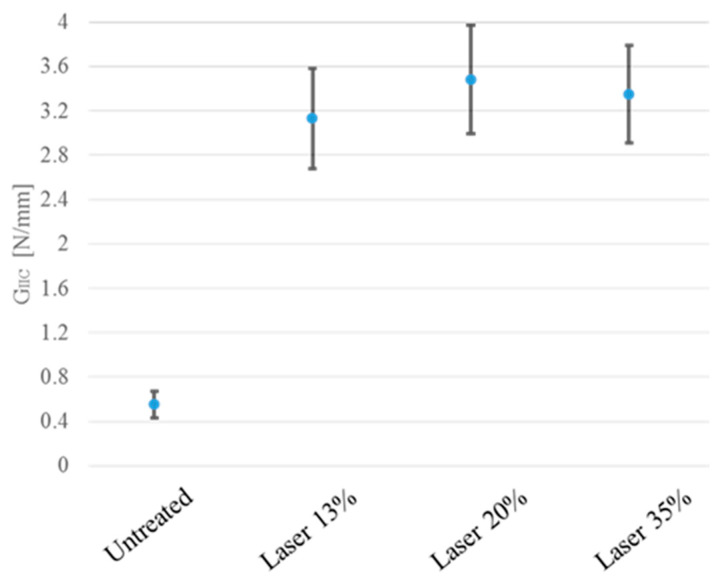
Fracture toughness obtained from experimental tests through the compliance beam method.

The difference of results between untreated and treated specimens became more pronounced when observing the fracture toughness obtained from experimental tests through the compliance beam method, as shown in Figure 6.

### 3.2. Numerical Results

For the parameters identification, the value of the fracture energy was directly obtained from the experimental tests, while the other parameters were obtained by fitting the experimental and numerical load-displacement curves for each density of treatment.

The cohesive numerical model allows to simulate failure in the location where the interface elements are defined. For this reason, the bonded joint can numerically fail only at the adherend/adhesive interface. The densities of treatment that allowed the failure in such a position were 0% and 13%, so it is possible to simulate only these densities of treatment.

Higher densities of treatments (20% and 35%) brought the failure of the adherends, not of the adherend/adhesive interface. The results related to these values were not quantitatively correlated to the increase of performance of the joint due to the laser treatment. In fact, the pretreatment allowed a resistance of the adherend/adhesive interface higher than the flexural resistance of the adherends, so the obtained values of these densities were representative of the adherend properties, not of the interface. For these reasons, the numerical model considered of only densities of treatment of 0% (untreated) and 13% (treated).

#### 3.2.1. Exponential Law

**Figure 7:**
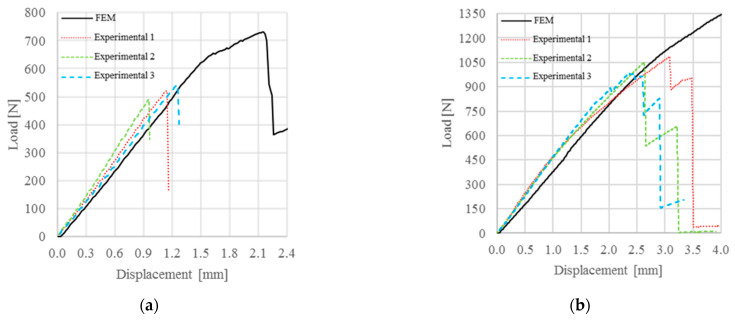
Comparison between experimental and numerical P-δ curve obtained with the exponential law for: (**a**) untreated specimens; (**b**) 13% treated specimens.

As it was defined, the exponential law did not allow the interface elements to have a rigid behavior during the elastic part of the cohesive law. In consequence, the interface presented an elastic deformation before the damage growth. In fact, the exponential law was recommended in case of interface elements with a finite thickness, and the determination of the cohesive parameters should be as a function of the elements thickness and the mechanical characteristics of the adhesive. Regarding the simulation of treated specimens, the exponential law did not allow forecasting of the specimens’ behavior because the fracture process zone appeared to be too extensive for numerically breaking the specimen, as shown in Figure 7. In fact, the main reason why this occurs is essentially related to the impossibility to subdivide the elastic strain and the plastic strain in the exponential law (due to the damaging of the elements). For this reason, the interface elements will always show a partially elastic deformation during the application of the load. This response generates an error in the prediction of the failure load, which is directly proportional to the applied load. It is likely that the error using the exponential law was such that the model was not able to predict the failure load of treated specimens.

Therefore, the exponential law appeared to be unsuitable for modelling the damage initialization and growth in this work, where the interface elements had no thickness and the adhesive was modelled with elastic behavior.

#### 3.2.2. Bilinear Law

The presence of elastic elements for the adhesive discretization allowed the use of interface elements with no thickness, which, in the case of bilinear law, should not have the elastic part of the law. In order to avoid convergence problems of the solution, a default value of *v_c_* of 10^−6^ was used. In fact, the adopted approach did not need the elastic mechanical response of the interface elements because this was represented by the mechanical response of the adhesive’ elements. For this reason, the value of *v_c_* should have been equal to zero. However, a value of *v_c_* equal to zero would have generated convergence problems to the simulation. For this reason, the chosen default value was equal to 1.00 nm: it did not represent a measured value, rather a parameter necessary to resolve convergence problems, avoiding elastic strains of the interface elements. The value of the fracture energy *G_c_* came from the experimental tests, while the maximum displacement *v_m_* represented the variable to get through the inverse method. As a result of this, the stiffness of the interface elements can vary as a function of the optimal value of *v_m_*, although their global behavior could well represent a rigid body with respect to the compliance of the adhesive elements. The cohesive parameters obtained from an inverse method for the bilinear law are reported in Table 6.

#### 3.2.3. Linear-Exponential Law

The considerations for the elastic part of the bilinear law were the same for the linear-exponential law. Specifically, for the linear-exponential law, it was possible to define a value of *v_c_* as small as possible, so that the mechanical response of the joint in the undamaged state depended only on the deformation adherend and adhesive elements. For that reason, a default value of *v_c_* of 10^−6^ was adopted, so the variable to optimize was the decay factor *q*. The cohesive parameters obtained from the inverse method for the linear exponential law are reported in Table 6.

### 3.3. Comparison between the Numerical and Experimental Results

Between the three chosen cohesive laws, only the exponential law showed some difficulties inherent in the numerical failure of ENF specimens, as shown in Figure 7. In particular, for untreated specimens, the failure appeared with a higher value of load and punch displacement, while treated specimens did not show failures.

**Figure 8:**
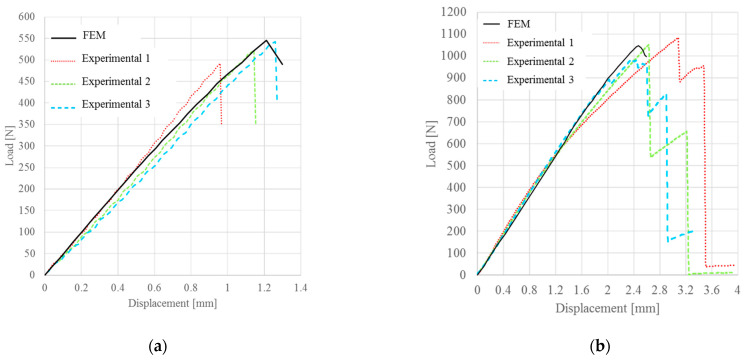
Comparison between experimental and numerical P-δ curve obtained with the bilinear law for: (**a**) untreated specimens; (**b**) 13% treated specimens.

In the case of bilinear law, the numerical results obtained were in good agreement with the experimental results, as shown in Figure 8. This type of cohesive law tended to overstate the critical displacement for untreated specimens and underestimate the critical displacement for treated specimens. However, these variations of the numerical response, with respect to the average behavior of the specimens, was about 5% and therefore negligible due to the dispersion of experimental results of the same magnitude (Table 6).

**Figure 9:**
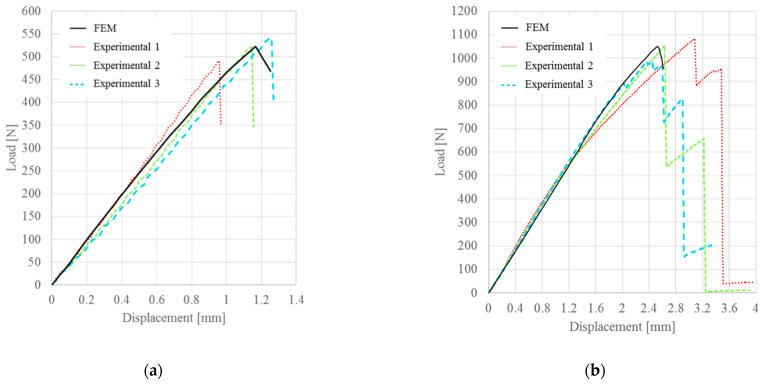
Comparison between experimental and numerical P-δ curve obtained with the linear-exponential law for: (**a**) untreated specimens; (**b**) 13% treated specimens.

The numerical results obtained using the linear-exponential law showed a slightly lower deviation of the critical displacement, with respect to the bilinear law, as illustrated in Figure 9. In particular, the obtained failure loads were very similar to the experimental loads, while the deviation of the critical displacement was about 5%. However, this value was lower, with respect to the results related to the bilinear law and lower than the experimental dispersion (Table 7).

The obtained results indicate that the linear exponential was more suitable for extending the model to a 3D approach. Further studies are needed to investigate how the use of local variation of properties of interface elements in a 3D model using a linear exponential could influence the numerical results in comparison with the experimental results. The aim will be to study the effects of each single dimple on the mechanical resistance of the joint: in this way, it will be possible to design the laser treatment in order to optimize the time and costs process.

## 4. Conclusions

An effectiveness of a CO_2_ laser texturing on the mechanical performance of ENF bonded joints, made in CFRP and epoxy adhesive, was investigated. The texturing was defined through dimples in a grid square, and the density of treatment was defined depending only on the grid dimension. An average increase of failure load was observed from 518.5 N, in the case of untreated specimens, to a range between 984.7 N and 1112.2 N in the case of treated specimens with different densities of treatment. The failure modes were observed, and a limit condition to evaluate the influence of the treatment was identified with a 13% of treated area. A cohesive model was developed to forecast the effect of the laser texturing, and three shapes of cohesive law were investigated: an exponential law, a bilinear law, and a linear exponential law. It was stated that the exponential law was not able to predict the failure load in the proposed approach, while the bilinear law revealed a good agreement between experimental and numerical results, with a forecast of the critical displacement of about 7% difference, with respect to the experimental average value. However, that difference was consistent with the dispersion obtained from the experimental tests. The linear exponential law revealed a better agreement with the experimental results. In the future, this activity should include further analyses to evaluate the effect of the laser texturing under mixed-mode conditions and, in particular, to evaluate the effect of the single dimple on the mechanical response of the bonded joint with more complex joint configurations. The results of this work represent a fundamental step to develop a 3D numerical tool for designing the optimal laser texturing as a function of the state of stress generated during the working life of the bonded joint, reducing costs and time of the pre-treatment process.

## Figures and Tables

**Table 1:** Mechanical properties of the adopted prepreg in cured state.

Properties	Value	
Young’s modulus, E_11_	133,800	MPa
Young’s modulus, E_22_ = E_33_	5900	MPa
Shear modulus, G_12_ = G_13_	12,000	MPa
Shear modulus, G_23_	2360	MPa
Poisson’s ratio, ν_12_ = ν_13_	0.26	
Poisson’s ratio, ν_23_	0.25	

**Table 2:** Laser parameters for realizing dimples, obtained from preliminary tests.

Parameters	Value	
Power	11.3	W
Wavelength	10,600	nm
Frequency	25	kHz
Scanning speed	5950	mm/s
Pulse duration	18	μs
Mode	TEM00	

**Table 3:** Experimental plan.

Factors	Number of Levels	Levels
Density of treatment (*ρ*)	4	0%; 13%; 20%; 35%
Repetitions	3	

**Table 4:** Mechanical properties of EA 9309.3 NA epoxy adhesive [29].

Factors	Value	
Tensile Strength	32.2	MPa
Tensile Modulus	2303	MPa
Shear Modulus	841	MPa
Poisson Ratio	0.36	
Elongation at break	10%	

**Table 5:** Critical loads obtained from experimental tests.

Treatment	N° Specimen	P_c_ [N]	Average [N]	St. Dev [N]	Coefficient of Variation (CV)
Untreated	1	521.5	518.5	25.6	4.94%
	2	491.5			
	3	542.4			
Treated (13%)	1	1083.6	1040.4	50.6	4.86%
	2	1052.9			
	3	984.7			
Treated (20%)	1	1036.5	1086.0	42.9	3.95%
	2	1109.3			
	3	1112.2			
Treated (35%)	1	1032.2	1046.6	45.3	4.33%
	2	1097.3			
	3	1010.2			

**Table 6:** Cohesive parameters obtained for bilinear and linear-exponential law.

Factors	Bilinear Law		Linear-Exponential Law	
	Untreated	Treated	Untreated	Treated
*t_n_* [MPa]	7.1	17.2	6.9	19.1
*v_c_* [mm]	1.00 × 10^−6^	1.00 × 10^−6^	1.00 × 10^−6^	1.00 × 10^−6^
*v_m_*	0.17	0.348	-	-
*q*	-	-	1.15 × 10^−5^	6.35 × 10^−6^
*G*_IIc_ [N/mm]	0.6	3.0	0.6	3.0

**Table 7:** Comparison between experimental results and numerical results.

		P_c_ [N]	Experimental Average Value	Experimental dev. st.	FEM	Difference from Experiments
Bilinear law	Untreated	P_C_ [N]	518.5	25.6	543.1	+4.74%
		δ_C_ [mm]	1.15	0.15	1.21	+5.21%
	Treated	P_C_ [N]	1040.4	50.6	1036.8	−0.35%
		δ_C_ [mm]	2.70	0.36	2.51	−7.04%
Linear exponential Law	Untreated	P_C_ [N]	518.5	25.6	522.6	+0.67%
		δ_C_ [mm]	1.13	0.15	1.17	+3.54%
	Treated	P_C_ [N]	1040.4	50.6	1040.9	+0.04%
		δ_C_ [mm]	2.70	0.36	2.56	−5.18%
